# Strain-Specific Virolysis Patterns of Human Noroviruses in Response to Alcohols

**DOI:** 10.1371/journal.pone.0157787

**Published:** 2016-06-23

**Authors:** Geun Woo Park, Nikail Collins, Leslie Barclay, Liya Hu, B. V. Venkataram Prasad, Benjamin A. Lopman, Jan Vinjé

**Affiliations:** 1 Division of Viral Diseases, Centers for Disease Control and Prevention, Atlanta, GA, United States of America; 2 Atlanta Research and Education Foundation (AREF), Atlanta, GA, United States of America; 3 Verna Marrs McLean Department of Biochemistry and Molecular Biology, Baylor College of Medicine, Houston, TX, United States of America; Tulane University, UNITED STATES

## Abstract

Alcohol-based hand sanitizers are widely used to disinfect hands to prevent the spread of pathogens including noroviruses. Alcohols inactivate norovirus by destruction of the viral capsid, resulting in the leakage of viral RNA (virolysis). Since conflicting results have been reported on the susceptibility of human noroviruses against alcohols, we exposed a panel of 30 human norovirus strains (14 GI and 16 GII strains) to different concentrations (50%, 70%, 90%) of ethanol and isopropanol and tested the viral RNA titer by RT-qPCR. Viral RNA titers of 10 (71.4%), 14 (100%), 3 (21.4%) and 7 (50%) of the 14 GI strains were reduced by > 1 log_10_ RNA copies/ml after exposure to 70% and 90% ethanol, and 70% and 90% isopropanol, respectively. RNA titers of 6 of the 7 non-GII 4 strains remained unaffected after alcohol exposure. Compared to GII strains, GI strains were more susceptible to ethanol than to isopropanol. At 90%, both alcohols reduced RNA titers of 8 of the 9 GII.4 strains by ≥ 1 log_10_ RNA copies/ml. After exposure to 70% ethanol, RNA titers of GII.4 Den Haag and Sydney strains decreased by ≥ 1.9 log_10_, whereas RNA reductions for GII.4 New Orleans strains were < 0.5 log_10_. To explain these differences, we sequenced the complete capsid gene of the 9 GII.4 strains and identified 17 amino acid substitutions in the P2 region among the 3 GII.4 variant viruses. When comparing with an additional set of 200 GII.4 VP1 sequences, only S310 and P396 were present in all GII.4 New Orleans viruses but not in the ethanol-sensitive GII.4 Sydney and GII.4 Den Haag viruses Our data demonstrate that alcohol susceptibility patterns between different norovirus genotypes vary widely and that virolysis data for a single strain or genotype are not representative for all noroviruses.

## Introduction

Noroviruses are the leading cause of epidemic and endemic gastroenteritis in people of all ages worldwide [1, 2]. The virus is transmitted directly from person to person or indirectly through the consumption of contaminated food, water, or by contact with contaminated surfaces and fomites [3–6]. The majority of norovirus outbreaks occur in semi-closed communities such as long-term care facilities, hospital wards, cruise ships, military barracks, and child-care centers where food is rarely implicated as the cause of the outbreak [6–9]. Increasing evidence shows that transmission via contaminated surfaces and hands plays a significant role in the spread of the virus [3]. Thus, cleaning and disinfection of surfaces and hands are likely the most effective ways to control norovirus gastroenteritis [10–12]. Washing hands with water and soap is recommended as the primary mode of intervention, whereas alcohol-based hand sanitizers (ABHS) can be used as an adjunct [13, 14]. Interpretation of efficacy data of ABHS on noroviruses is challenging, because their effects do not necessarily depend on alcohol but rather on the formulation of the product [15, 16].

Alcohol induced virolysis of non-enveloped viruses is characterized by destruction or serious damage of viral capsids, which results in leakage of viral RNA a process referred to as virolysis [16, 17]. RNase pre-treatment of viruses prior to extracting RNA and testing by RT-qPCR has become the standard method to remove leaked viral RNA, which is considered a proxy for measuring remaining infectivity as in principle only viral genomes protected by an intact viral capsid are enumerated [18, 19]. Such correlation between reduced viral RNA titers and loss of infectivity after alcohol treatment has been demonstrated for murine norovirus [16].

Most human norovirus infections are caused by viruses that belong to genogroup GI and GII viruses. Of these, GII.4 viruses have caused the majority of the infections over the last decade [2, 20]. The RNA genome of noroviruses consists of three open reading frames (ORF). ORF1 encodes six nonstructural proteins whereas ORF2 and ORF3 encode the major capsid protein (VP1) and minor capsid protein (VP2). VP1 consists of a shell domain (S) and a protruding domain (P), with is further divided into two subdomains (P1 and P2) that determine the antigenicity of the virus. The P2 domain of VP1 is the area of the capsid with the most interaction with the host cells [21] and crystallography studies of norovirus *in vitro* have demonstrated that the P2 domain binds to histoblood group antigens for which it has highly conserved binding pockets. Deletion of just one amino acid from one of the P2 epitopes has been shown to eliminate the binding capability of norovirus virus-like-particles (VLPs)[22]. We recently reported distinct different virolysis patterns for GI.5 and GII.13 viruses after exposure to alcohol based on which we hypothesized that multiple different virolysis patterns among viruses from different norovirus genotypes exist [23]. Based on these data, we tested this hypothesis by analyzing the susceptibility of a panel of human norovirus strains including different GI and GII genotypes against ethanol and isopropanol. Among the GII.4 viruses, GII.4 New Orleans viruses but not GII.4 Den Haag and GII.4 Sydney viruses were resistant to ethanol. Further evaluation of 200 GII.4 VP1 sequences generated in this study, indicated that two protruding amino acid substitutions present on the P domain of the VP1 may be associated with resistance to ethanol.

## Materials and Methods

### Viruses

Norovirus positive stool specimens (14 GI and 16 GII) were obtained from acute gastroenteritis outbreaks that were submitted to CDC from 2010 to 2013. Additionally, three stool samples from human volunteers (GI.1 viruses) were kindly provided by Dr. Christine Moe at Emory University. A 10% suspension of each stool sample was made in phosphate-buffered saline (PBS; pH 7.5) and clarified by centrifugation at 11, 337×g (13,000 rpm) for 10 min. The clarified supernatant was then filtered through a 0.45-μm pore size Millex-MP filter (Millipore, Billerica, MA) to remove solid particles. Each filtered sample was stored at -80°C until use. To minimize the possible effect of individual stool matrix, each norovirus strain was pooled separately with three strains from a different genogroup. For example, three pools of the same GI strain were prepared each mixed with a different GII virus.

### Suspension test

Different concentrations (50%, 70%, and 90% (v/v)) of ethanol and isopropanol (Sigma-Aldrich, St Louis, MO) were prepared in distilled water. A suspension test was used to measure the efficacy of different alcohols against human noroviruses as described previously[16]. Briefly, 450 μl of alcohol (or PBS as negative control) was mixed with 50 μl of virus followed by incubation for 1 minute at room temperature. Of the alcohol-virus mixture, 100 μl was transferred into 900 μl of 10% fetal bovine serum to stop the effect of alcohol on the virus. Samples were then immediately subjected to RNase treatment followed by RNA extraction.

### RNase treatment, RNA extraction and TaqMan real-time RT-PCR

To remove viral RNA from damaged viral packages, 100 μl of neutralized alcohol (or PBS)-treated pooled GI/GII samples were incubated with 33 μl of DI water, 1μl of RNase One^TM^ Ribonuclease and 15 μl of 10×RNAse buffer (Promega, Madison, WI) for 1 h at 37°C. After stopping the reaction was stopped by adding 2 μl of RNase inhibitor (Invitrogen, Carlsbad, CA) [24], viral RNA was extracted using the MagMAX™—96 Viral RNA Isolation Kit (Ambion) on the KingFisher® instrument. Viral RNA was quantified by norovirus TaqMan realtime RT-qPCR as previously described [16, 25]. Untreated GI/GII samples were used to determine the initial RNA titer of each GI and GII strain in each test. Standard curves of norovirus GI.7 and GII.12 RNA transcripts were included to ensure quality control across different experiments [26] and to convert Ct values into RNA copy numbers.

### ORF2 sequencing of GII.4 viruses

To identify amino acids that possibly could explain why GII.4 New Orleans viruses, and not the genetically closely-related GII.4 Den Haag and GII.4 Sydney viruses, were resistant to ethanol, we sequenced the complete ORF2 gene of 8 of the 9 GII.4 viruses that were included in the panel. For one virus strain (2010746526), no sufficient sample was left to obtain the complete ORF2 sequence. In addition, complete ORF2 sequence of an additional 200 GII.4 viruses from norovirus outbreaks in the US that occurred between 2009–2016 were determined [27]. ORF2 sequences were amplified using oligonucleotide primer PanGIIR1 (5’- GTC CAG GAG TCC AAA A-3) and forward primer ring2 (5’- TGG GAG GGC GAT CGC AAT CT-3’) using long RT-PCR using the Phusion PCR Kit with the addition of 3% dimethyl sulfoxide (Finnzymes, Woburn, MA, USA) as described previously [28]. The GenBank accession numbers for the strains sequenced in this study are: KX371603-KX371610 and KX353947-KX354146).

The deduced amino acid sequences of the VP1 sequences including reference GII.4 viruses GII.4 Den Haag (JN400607), GII.4 New Orleans (GV445325), and GII.4 Sydney (JX459908) were then aligned using Clustal W. The alignment was then mapped onto the X-ray structure of the P domain of a representative GII.4 virus (PDB ID: 3SLD) using Chimera software [29] to infer their locations of the amino acids in the P2 subdomain structure and examine their solvent accessibility.

### Statistical analysis

Reduction of viral RNA reduction was determined by calculating the log_10_ (N_d_/N_0_), where N_0_ is the number of RNA copies detected in untreated samples and N_d_ is the number of viral particles or RNA copies in the alcohol-treated samples. TaqMan real-time RT-PCR data were expressed as the mean of at least three replicates from each independent experiment. Univariate linear regression models were fitted to compare overall virolysis patterns between norovirus strains, and the Mann-Whitney test was used to determine significant differences in RNA reduction levels using PASW Statistic 18 software (IBM SPSS Inc, New York, NY) [30]. P values of < 0.05 were considered statistically significant.

### Ethics Statement

Stool specimens from human volunteers without any personal identifying information that had been determined IRB-exempt were used in this study.

## Results

### Virolysis patterns of GI norovirus strains after exposure to ethanol and isopropanol

Overall GI viruses were more sensitive to ethanol than to isopropanol which was most apparent for 70% and 90% solutions, although 2 GI.6 and 1 GI.7 strains were equally sensitive to ethanol and isopropanol. Exposure to 70% and 90% ethanol reduced viral RNA titers of 9 and 13 of the 14 GI strains by > 1.8 log_10_ RNA copies/ml, respectively. The titers of 4 (3 GI.6 and 1 GI.7) of the GI strains were > 1.8 log_10_ RNA copies/ml reduced after exposure to 90% isopropanol, whereas no RNA reduction was observed for 50% ethanol, and for 50% and 70% isopropanol (Table 1). Overall, 70% or 90% ethanol and 90% isopropanol reduced RNA titers of GI strains (P<0.001) with an average of 1.8 ± 1.1, 2.6 ± 1.0, and 1.0 ± 1.0 log_10_ RNA copies, respectively.

**Table 1:** Virolysis patterns of GI norovirus strains after exposure to ethanol and Isopropanol.

			Reduction in RNA titer (log_10_ RNA copies/ml)^1^					
Genogroup	Strain ID	Genotype	50% Ethanol	70% Ethanol	90% Ethanol	50% Isopropanol	70% Isopropanol	90% Isopropanol
GI	8FIIb (4–3)	GI.1	0.1 ± 0.2	2.5 ± 0.7	2.6 ± 0.7	0.0 ± 0.4	0.1 ± 0.5	0.4 ± 0.4
	17–6	GI.1	0.1 ± 0.4	1.9 ± 0.0	1.8 ± 0.1	0.0 ± 0.1	0.0 ± 0.1	0.0 ± 0.2
	34–6	GI.1	0.0 ± 0.4	2.0 ± 0.3	1.2 ± 0.2	0.0 ± 0.4	0.0 ± 0.3	0.0 ± 0.5
	2010746440	GI.2	0.0 ± 0.3	0.5 ± 0.1	3.9 ± 0.1	0.0 ± 0.2	0.7 ± 0.6	0.7 ± 0.6
	2010746435	GI.3b	0.1 ± 0.2	0.4 ± 0.3	3.8 ± 0.1	0.0 ± 0.2	0.0 ± 0.2	1.4 ± 0.2
	2010746565	GI.3b	0.1 ± 0.1	0.2 ± 0.2	2.1 ± 0.2	0.0 ± 0.1	0.0 ± 0.2	1.7 ± 0.6
	2013775245	GI.3b	0.0 ± 0.3	3.5 ± 0.7	3.5 ± 0.7	0.2 ± 0.1	0.0 ± 0.2	0.1 ± 0.2
	2013843465	GI.3b	0.4 ± 0.7	2.9 ± 0.5	3.2 ± 0.9	0.5 ± 0.7	0.4 ± 0.7	0.8 ± 0.5
	2010746333	GI.6	0.0 ± 0.0	> 1.8	> 1.8	0.2 ± 0.2	0.8 ± 0.7	> 1.8
	2013751712	GI.6	0.0 ± 0.2	> 1.9	> 1.9	0.0 ± 0.1	0.6 ± 0.1	> 1.9
	2013751688	GI.6	1.2 ± 0.4	2.3 ± 0.5	2.4 ± 0.5	0.3 ± 0.1	1.5 ± 0.6	2.3 ± 0.3
	2013751693	GI.6	0.5 ± 0.5	2.1 ± 0.4	2.0 ± 0.2	0.0 ± 0.4	1.4 ± 0.5	1.4 ± 0.5
	2010746327	GI.7	0.1 ± 0.1	1.0 ± 0.5	3.4 ± 0.3	0.5 ± 0.1	1.6 ± 0.3	3.4 ± 0.3
	2011755567	GI.7	0.0 ± 0.1	0.2 ± 0.1	3.3 ± 0.7	0.0 ± 0.1	0.0 ± 0.2	0.4 ± 0.2
	Average reduction		0.9 ± 0.9	1.2 ± 1.1	1.4 ± 0.9	0.6 ± 0.7	0.3 ± 0.4	1.0 ± 0.8

^1^: Reduction in RT-qPCR titer is expressed as the mean ± standard deviation of four replicates from two independent experiments

**Fig 1:**
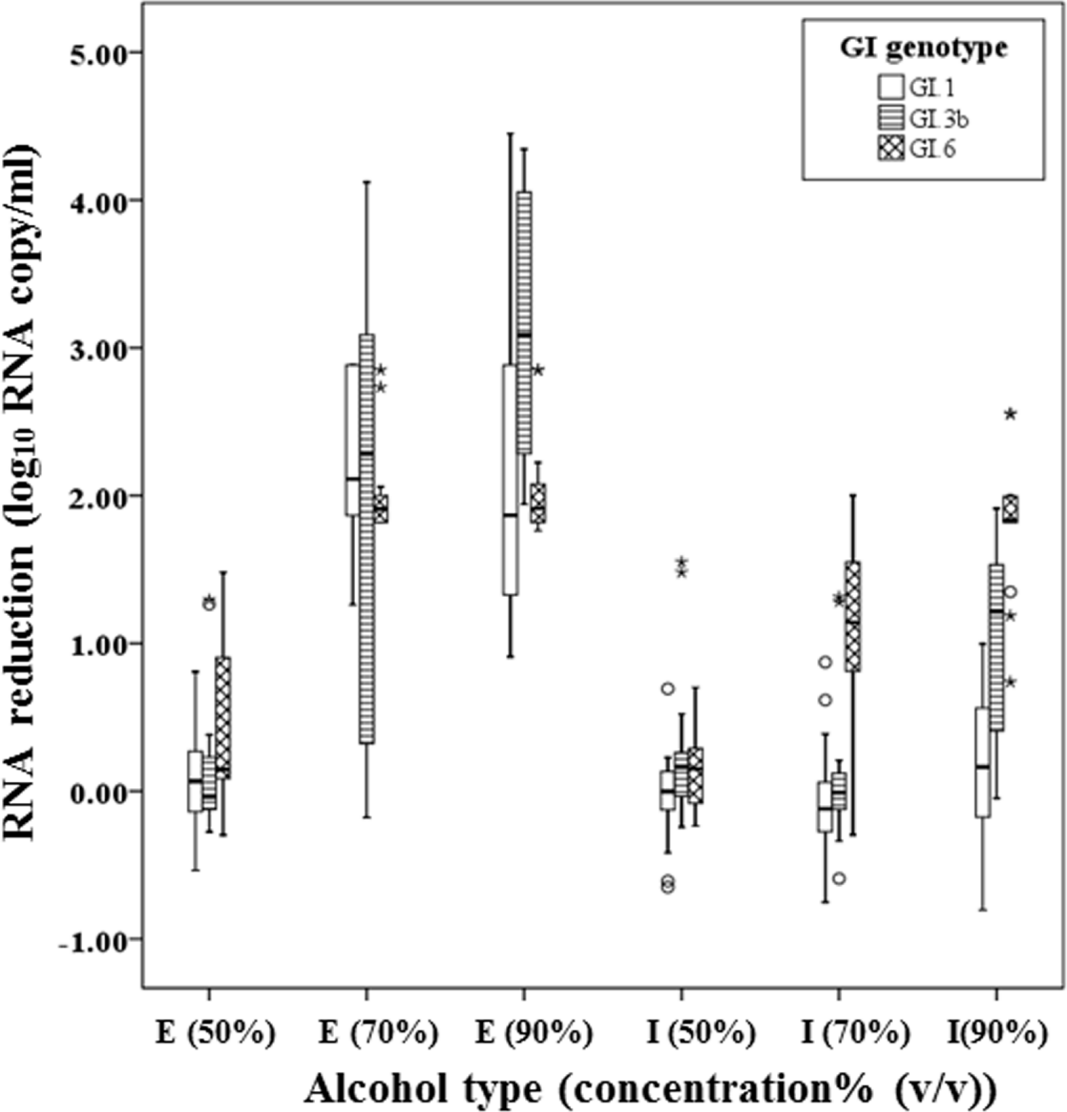
Virolysis patterns of GI.1, GI.3b and GI.6 norovirus strains after exposure to ethanol (E) and isopropyl alcohol (I) at 3 different concentrations (50%, 70%, and 90%). 3 GI.1, 4 GI.3b and 4 GI.4 strains with three replicate per each were consolidated by genotype and were expressed as a box plot. The upper, lower ends of the box and the horizontal line in the box indicate the first (Q1), third quantiles (Q3) and median value of all data, respectively. The lower and higher ends of whiskers indicate the minimum and maximum value of all data, respectively. Isolated data points are outliers.

The median RNA reduction levels of GI.1, GI.3b and GI.6 viruses were 0.16, 1.2 and 1.8 log_10_ RNA copies/ml after exposure to 90% isopropanol, respectively (Fig 1). When exposed to 90% ethanol, median viral reductions of GI.1 and GI.6 viruses were lower compared to GI.3b viruses, whereas GI.6 viruses were more sensitive against 70% isopropanol than GI.1 and GI.3b viruses (P < 0.05). However, all GI strains showed similar RNA reductions after exposure to 50% and 70% ethanol and 50% isopropanol.

### Virolysis patterns of GII norovirus strains after exposure to ethanol and isopropanol

After exposure to alcohol, RNA titers of 6 of the 7 non-GII.4 strains were unaffected (≤ 0.8 log_10_ RNA copies) except for one GII.12 strain, of which titer was reduced by 1.4 log_10_ RNA copies/ml. Exposure to 90% alcohols reduced RNA titers of all 9 GII.4 strains by ≥ 0.9 log_10_ RNA copies/ml. Overall, exposure to 70% ethanol as well as 90% ethanol and 90% isopropanol resulted in significant RNA reductions for GII strains (P < 0.001) with an average of 1.2 ± 1.1, 1.4 ± 0.9, and 1.0 ± 0.8 log_10_ RNA copies, respectively.

After exposure to 50% and 70% ethanol, RNA titers of GII.4 Den Haag (n = 2) and GII.4 Sydney (n = 4) viruses were reduced by >1.9 log10 RNA copies/ml whereas the titers for GII.4 New Orleans (n = 3) viruses were reduced by less than 0.5 log_10_ RNA copies/ml (Table 2). After exposure to 50% isopropanol, RNA titers of both GII.4 Den Haag viruses and 3 of the 4 GII.4 Sydney viruses were reduced by > 1.0 log_10_ RNA copies/ml, while RNA titers of GII.4 New Orleans were reduced by ≤ 0.5 log_10_ RNA copies/ml (Table 2).

**Table 2:** Virolysis patterns of GII norovirus strains after exposure to ethanol and isopropanol.

-			Reduction in RNA titer (log_10_ RNA copies/ml)^1^					
Genogroup	Strain ID	Genotype	50% Ethanol	70% Ethanol	90% Ethanol	50% Isopropanol	70% Isopropanol	90% Isopropanol
GII	2010746610	GII.1	0.2 ± 0.1	0.3 ± 0.2	0.7 ± 0.3	0.1 ± 0.1	0.1 ± 0.1	0.4 ± 0.1
	2010746618	GII.2	0.4 ± 0.2	0.4 ± 0.2	0.7 ± 0.1	0.2 ± 0.1	0.2 ± 0.2	0.3 ± 0.2
	2013775428	GII.4^3^	2.3 ± 0.2	2.3 ± 0.3	2.4 ± 0.1	2.0 ± 0.5	0.9 ± 0.3	2.4 ± 0.1
	2013775418	GII.4^3^	1.2 ± 0.5	2.5 ± 0.5	2.7 ± 0.4	1.4 ± 0.3	0.9 ± 0.2	1.5 ± 0.4
	2010746350	GII.4^4^	0.4 ± 0.1	0.5 ± 0.2	1.4 ± 0.3	0.4 ± 0.1	1.0 ± 0.5	2.4 ± 0.4
	2010746173	GII.4^4^	0.2 ± 0.1	0.5 ± 0.4	0.9 ± 0.1	0.0 ± 0.2	0.0 ± 0.0	1.0 ± 0.3
	2010746526^2^	GII.4^4^	0.4 ± 0.2	0.4 ± 0.2	1.6 ± 0.3	0.5 ± 0.2	0.5 ± 0.1	1.5 ± 0.1
	2013775232	GII.4^5^	2.0 ± 0.3	2.1 ± 0.1	2.2 ± 0.1	0.2 ± 0.1	0.1 ± 0.2	2.1 ± 0.6
	2013751795	GII.4^5^	1.1 ± 0.2	2.2 ± 0.5	1.6 ± 0.2	1.0 ± 0.2	0.5 ± 0.3	0.9 ± 0.3
	2013843445	GII.4^5^	1.8 ± 0.8	1.9 ± 0.7	2.4 ± 0.2	1.1 ± 0.3	0.4 ± 0.3	1.8 ± 0.2
	2013843449	GII.4^5^	2.5 ± 0.4	3.0 ± 0.2	3.1 ± 0.3	1.8 ± 0.3	0.7 ± 0.1	1.7 ± 0.2
	2010746514	GII.7	0.1 ± 0.0	0.4 ± 0.1	0.7 ± 0.1	0.1 ± 0.1	0.1 ± 0.1	0.8 ± 0.8
	2012706250	GII.12	0.0 ± 0.1	0.1 ± 0.1	0.7 ± 0.1	0.0 ± 0.2	0.0 ± 0.1	0.0 ± 0.2
	2010746037	GII.12	0.0 ± 0.2	0.0 ± 0.1	0.2 ± 0.2	0.0 ± 0.2	0.0 ± 0.1	0.0 ± 0.1
	2010746401	GII.12	0.3 ± 0.3	1.4 ± 0.1	1.6 ± 0.1	0.2 ± 0.1	0.0 ± 0.2	0.4 ± 0.1
	2010746174	GII.13	0.3 ± 0.1	0.4 ± 0.1	0.2 ± 0.2	0.5 ± 0.2	0.5 ± 0.1	0.0 ± 0.2
	Average reduction		0.9 ± 0.5	1.2 ± 1.1	1.4 ± 0.9	0.6 ± 0.7	0.3 ± 0.4	1.0 ± 0.8

^1^: Reduction in RT-qPCR titer is expressed as the mean ± standard deviation of four replicates from two independent experiments

^2^: Sample was depleted and no full ORF2 sequence was obtained

^3^: GII.4 Den Haag

^4^: GII.4 New Orleans

^5^: GII.4 Sydney

**Fig 2:**
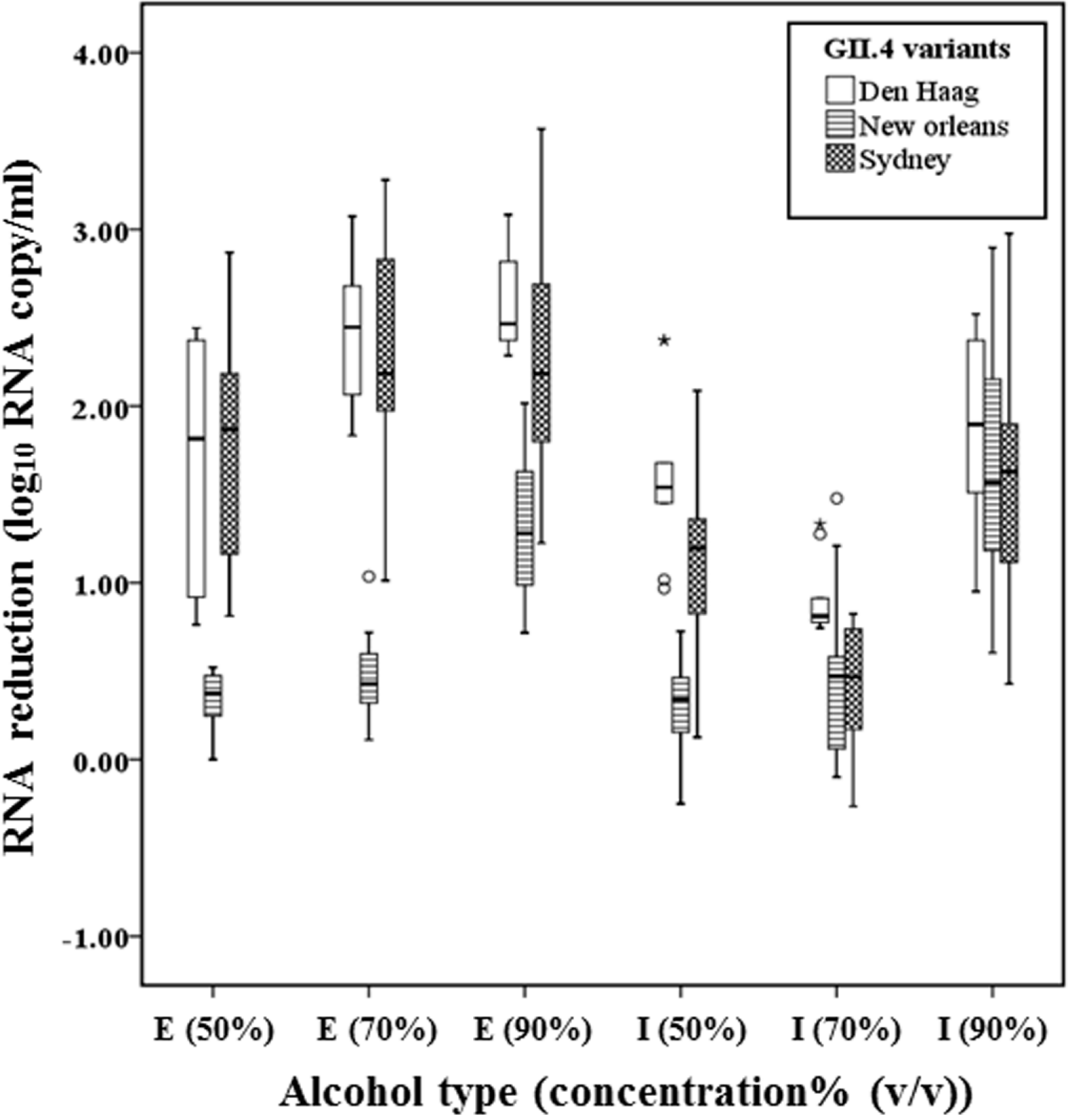
Virolysis patterns of GII.4 variants including GII.4 Den Haag, GII.4 New Orleans and GII.4 Sydney viruses after exposure to ethanol (E) and isopropyl alcohol (I) at 3 different concentrations (50%, 70%, and 90%). 2 GII.4 Den Haag, 3 GII.4 New Orleans and 4 GII.4 Sydney strains with three replicates were consolidated by genotype and were expressed as a box plot. The upper, lower ends of the box and the horizontal line in the box indicate the first (Q1), third quantiles (Q3) and median value of all data, respectively. The lower and higher ends of whiskers indicate the minimum and maximum value of all data, respectively. Isolated points are outliers.

The median RNA reductions of the three types of GII.4 variant strains (Den Haag, New Orleans, Sydney strains) were not different after exposure to 90% isopropanol. However, after exposure to 50%,70%, 90% ethanol and 50% isopropanol, the median RNA reductions of GII.4 Den Haag and Sydney strains were significantly higher than for the GII.4 New Orleans viruses (P ≤ 0.001) (Fig 2). Interestingly, for 70% isopropanol, the median RNA titers were significantly more reduced for GII.4 Den Haag, compared to GII.4 Sydney strains (P < 0.001).

### Comparing amino acids of P2 subdomain of GII.4 Den Haag, GII.4 New Orleans, and GII.4 Sydney viruses

**Fig 3:**
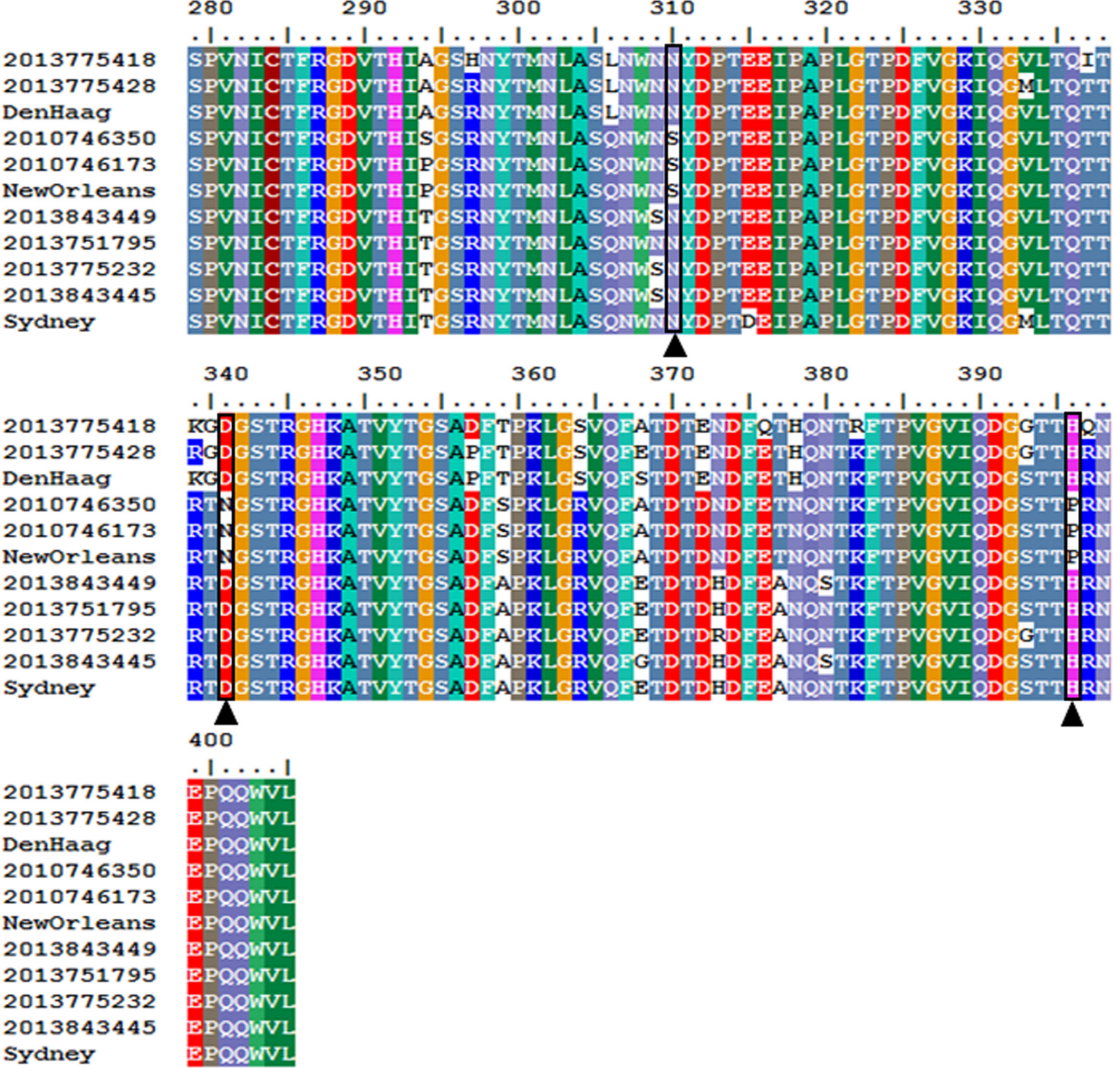
P2 subdomain (amino acids (aa) 279–405) sequence alignment of GII.4 Den Haag, GII.4 New Orleans, and GII.4 Sydney viruses. Key amino acid changes, S310N, andN341D, and P396H are indicated with black boxes.

**Fig 4:**
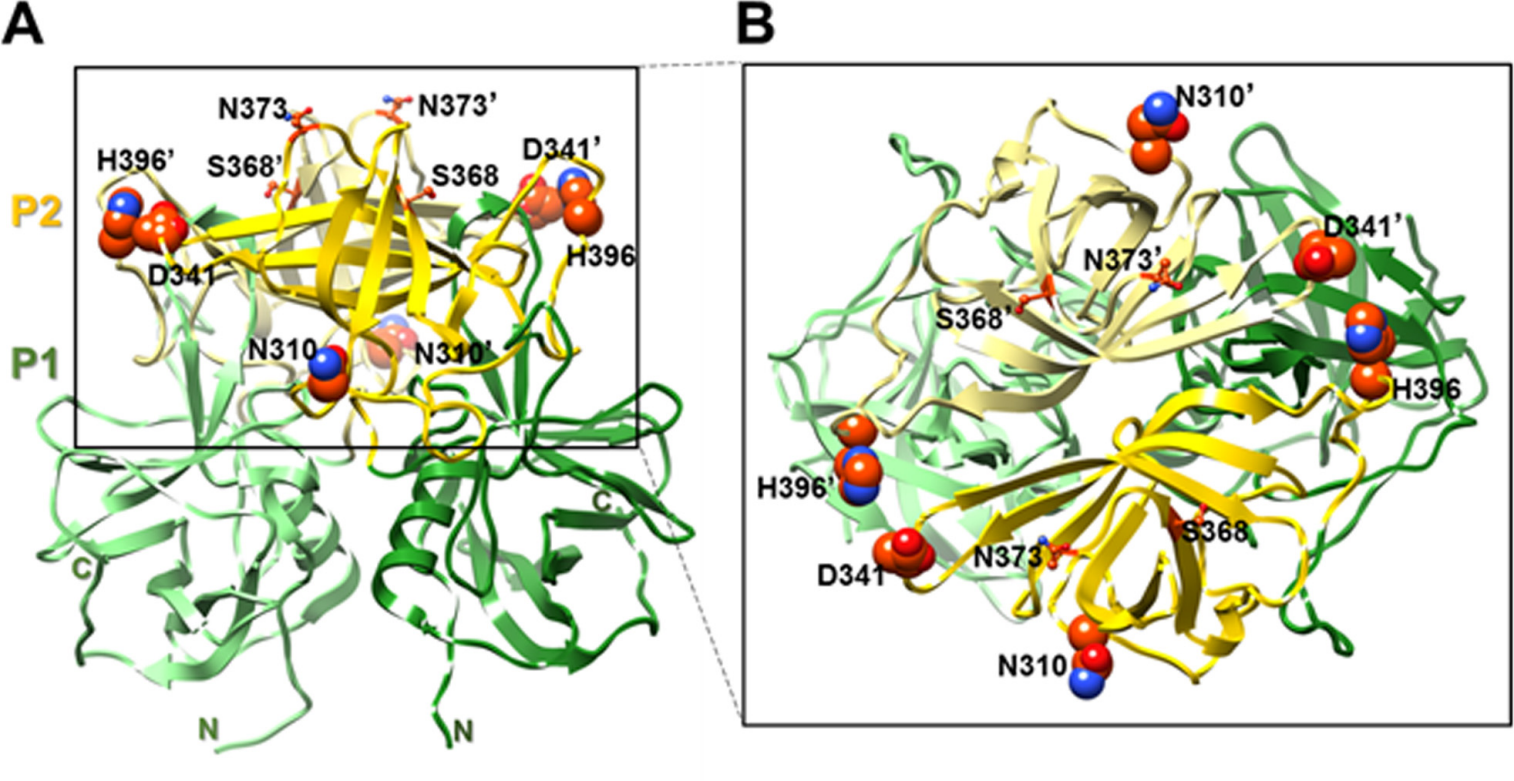
Mapping of amino acid (aa) sequence variations between strains on the P domain of capsid protein VP1. **(A)** Cartoon representation of the P domain of capsid protein VP1 dimer (side view) (PDB ID: 3SLD). The P1 (aa 225–278 and 406–519) and P2 (aa 279–405) subdomains are colored in green and yellow, respectively. Side chains of key aa are shown, with N310, H396 and D341 presented as spheres. The residues in the opposing subunit of the dimer are labeled ′. **(B)** Top view of the P-domain dimer. The two subunits are shown in yellow and light yellow, respectively. Amino acids are labeled as in (A).

We sequenced the complete capsid genes of 8 of 9 GII.4 viruses that were analyzed for their resistance to alcohols. The P1 and S domain had 4 and 0 amino acid changes, respectively, while the P2 region had 17 amino acid substitutions (Den Haag, New Orleans and Sydney) (Fig 3). Of those 17 amino acid changes in the P2 subdomain, three mutations (S310N, N341D and P396H) were consistently present in the two alcohol-sensitive GII.4 variants (Sydney and the Den Haag viruses), but not in the New Orleans GII.4 variant viruses (Fig 4). After analyzing the VP1 sequences from an additional 200 norovirus GII.4 viruses including 67 GII.4 New Orleans strains, 32 GII.4 Den Haag viruses and 101 GII.4 Sydney viruses, only amino acids S310 and P396 were conserved among all GII.4 New Orleans viruses while there were not present in Den Haag and Sydney viruses suggesting that these two amino acids may be associated with a higher resistance to alcohols.

## Discussion

Appropriate hand hygiene is the single most effective way to prevent norovirus transmission. In the laboratory, the effect of ABHS on norovirus has been studied primarily by using the Norwalk virus (GI.1) reference strain [15, 31]. We recently reported significant differences in sensitivity against ethanol between GI.5 and GII.13 noroviruses [23]. In the current study we demonstrated several different virolysis patterns among different GI and GII norovirus strains after exposure to ethanol and isopropanol each of which are active ingredients of ABHS [14]. Overall, all GI strains were more susceptible to ethanol than to isopropanol. Interestingly, of all GII.4 strains tested, only the GII.4 Den Haag and GII.4 Sydney viruses but not GII.4 New Orleans viruses were susceptible to ethanol. Thus, distinct differences in virolysis patterns among the three GII.4 variant viruses suggests that genetic similarity may not be the sole determinant of susceptibility to virolysis.

According to the Klein-DeForest scheme, a typical dose response relationship exists between the concentration of alcohol and the level of virus inactivation [32]. The type of alcohol may be a key factor influencing the degree of virolysis, as evidenced by a strong correlation between the hydrophobicity of short chain alcohols and their ability to alter the integrity of cell membranes [33]. Furthermore, ethanol is more hydrophilic than isopropanol and is more reactive against hydrophilic viruses [32], possibly explaining the virolysis patterns of GI viruses which were highly susceptible to ≥ 70% ethanol and varying but less susceptible to isopropanol. These different virolysis patterns of norovirus strains suggest that the dose response relationships for GII strains may be different from that of GI strains as well as other hydrophilic nonenveloped viruses such as poliovirus and Coxsackie B1 virus, which are susceptible to ethanol and isopropanol at concentrations of 70% or higher.

The P2 hypervariable subdomain of noroviruses is surface exposed and contains histoblood group antigen as well as important antigen binding sites that interact with the human host [34, 35]. We found that ethanol sensitive GII.4 strains such as GII.4 Den Haag and GII.4 Sydney have compared to GII.4 New Orleans viruses 3 surface-exposed amino acids (S310N, N341D and P396H) altered in the P2 region. To verify if these 3 amino acids were conserved among other GII.4 New Orleans viruses, we sequenced and analyzed VP1 sequences from 200 GII.4 strains that caused norovirus outbreaks in the US between 2009 ─ 2016. Interestingly, all 29 GII.4 New Orleans VP1 sequences had conserved S310 and P396 amino acids whereas the other 184 GII.4 VP1 sequences including GII.4 Den Haag and GII.4 Sydney viruses had the S310N and P396H substitutions. The N341 amino acid was not conserved among the 29 VP1 GII.4 New Orleans sequences and therefore was considered not directly involved in the resistance against GII.4 New Orleans viruses to alcohols. The S310 and P396 amino acids are located in close proximity of the epitopes involved in binding of the virus to histoblood group antigens and antibodies [34, 35]. Furthermore, compared to the two amino acids found in Den Haag and Sydney viruses, S310 and P396 are more hydrophilic facilitating interactions with water which favors reactivity with alcohols [36]. Thus, certain amino acid substitutions in the P2 domain likely alter electrostatic surface charges which perhaps make GII.4 Den Haag and GII.4 Sydney viruses, compared to GII.4 New Orleans viruses, more susceptible to alcohol.

The physical and chemical properties of viral capsid proteins may also affect interactions with environmental matrices which may lead to different environmental behaviors (transport, survival and adsorption) and different disinfection patterns [37, 38]. Reportedly, several non-GII.4 norovirus genotypes have been more often associated with foodborne norovirus outbreaks compared to outbreaks where person-to-person was the main transmission route [27], indicating that perhaps survival of non-GII.4 genotypes on food matrices may be dependent on the genotype. Additional proof that environmental survival is directly related to the composition of the viral capsid has been shown by the differences in susceptibility to alcohol, pH, chlorine, and other environmental stress factors of murine norovirus compared to feline calicivirus [16, 23, 39]. We speculate that strain-specific virolysis patterns observed after exposure to alcohol are likely associated with differences in the hydrophobicity (or hydrophilicity) of their capsid proteins.

In summary, we found that after exposure to 70% ethanol several norovirus GII.4 strains showed no reduction (< 0.5 log) in viral RNA titer whereas other norovirus GII.4 variants showed a 1.9–3 log reduction. Interestingly, GII.4 New Orleans viruses, which in the P2 domain differ only in 17 amino acids, compared to GII.4 Den Haag and GII.4 Sydney viruses, showed almost no reduction in viral RNA titers after exposure to alcohol. These differences in susceptibility correlated with the consistent presence of two amino acids S310 and P396 located on the protruding (P2) domain of the GII.4 New Orleans capsids. To confirm the importance of these amino acids among GII.4 viruses in protection against capsid degradation by alcohols, additional experiments, ideally using infectious clones to introduce specific amino acids in a backbone of a strain that is less sensitive to alcohol are required. Since the ratio between RNA reduction and infectivity reduction by alcohols remains unknown [16, 17], the ultimate assessment whether alcohols are capable of appropriately disinfecting human norovirus, will require confirmation in a cell culture system for human norovirus [40]. The maximum concentration of ethanol allowed in commercially available ABHS in the U.S is 70% which is also recommended by the WHO [14, 41]. Published data on the efficacy of ABHS against human norovirus are conflicting [42, 43] which, together with the findings in our study, illustrates that multiple norovirus genotypes should be used to assess the efficacy of ABHS.
